# Racine Medical Association

**Published:** 1863-01

**Authors:** 


RACINE MEDICAL ASSOCIATION.

The Racine Medical Association was organized on Thursday of last week, and the following officers elected:—President, J. L. Page, M. D.; Vice President, W. Wadsworth, M. D.; Secretary, J. G. Meacham, M. D.; Treasurer, P. R. Hay, M. D.; Librarian, O. Peak, M. D.

A tariff of prices was adopted to *match* the high price of living; which is the right and duty of every medical organization in the land.

I will give you a report from time to time of our proceedings, if anything worthy of record comes up.

Racine, Wis., Dec. 19. 1862. J. G M.

